# ADHD-200 Global Competition: diagnosing ADHD using personal characteristic data can outperform resting state fMRI measurements

**DOI:** 10.3389/fnsys.2012.00069

**Published:** 2012-09-28

**Authors:** Matthew R. G. Brown, Gagan S. Sidhu, Russell Greiner, Nasimeh Asgarian, Meysam Bastani, Peter H. Silverstone, Andrew J. Greenshaw, Serdar M. Dursun

**Affiliations:** ^1^Department of Psychiatry, University of AlbertaEdmonton, AB, Canada; ^2^Department of Computing Science, University of AlbertaEdmonton, AB, Canada; ^3^Alberta Innovates Centre for Machine LearningEdmonton, AB, Canada

**Keywords:** ADHD, children, classifier, diagnosis, functional connectivity, ICA, machine learning, multivoxel pattern analysis

## Abstract

Neuroimaging-based diagnostics could potentially assist clinicians to make more accurate diagnoses resulting in faster, more effective treatment. We participated in the 2011 ADHD-200 Global Competition which involved analyzing a large dataset of 973 participants including Attention deficit hyperactivity disorder (ADHD) patients and healthy controls. Each participant's data included a resting state functional magnetic resonance imaging (fMRI) scan as well as personal characteristic and diagnostic data. The goal was to learn a machine learning classifier that used a participant's resting state fMRI scan to diagnose (classify) that individual into one of three categories: healthy control, ADHD combined (ADHD-C) type, or ADHD inattentive (ADHD-I) type. We used participants' personal characteristic data (site of data collection, age, gender, handedness, performance IQ, verbal IQ, and full scale IQ), without any fMRI data, as input to a logistic classifier to generate diagnostic predictions. Surprisingly, this approach achieved the highest diagnostic accuracy (62.52%) as well as the highest score (124 of 195) of any of the 21 teams participating in the competition. These results demonstrate the importance of accounting for differences in age, gender, and other personal characteristics in imaging diagnostics research. We discuss further implications of these results for fMRI-based diagnosis as well as fMRI-based clinical research. We also document our tests with a variety of imaging-based diagnostic methods, none of which performed as well as the logistic classifier using only personal characteristic data.

## 1. Introduction

Attention deficit hyperactivity disorder (ADHD) is a psychiatric disorder characterized by impulsiveness, inattention, and hyperactivity. This condition affects about 5% of children and adolescents worldwide (Polanczyk et al., 2007). ADHD imposes substantial personal burdens on individuals as well as economic costs to society. The Diagnostic and Statistical Manual of Mental Disorders, Fourth Edition (DSM-IV-TR) identifies three subtypes of ADHD: ADHD hyperactive-impulsive subtype (ADHD-H), ADHD inattentive subtype (ADHD-I), and ADHD combined hyperactive-impulsive and inattentive subtype (ADHD-C).

Previous fMRI studies have investigated the neurobiology of ADHD (see King et al., 2003; Bush et al., 2005; Liston et al., 2011). The majority of such studies focused on group analyses, in which summary statistics computed across groups are compared to some baseline or to each other. For example, a given brain region's mean fMRI activation level taken across a group of ADHD patients might be compared to the equivalent mean activation level taken across a group of healthy control participants. Recent years have seen a growing interest in using patterns of fMRI data to distinguish individuals, not just groups. For example, machine learning (artificial intelligence) techniques have been used to diagnose various psychiatric illnesses based on an individual's fMRI data (Zhu et al., 2005, 2008; Shinkareva et al., 2006; Calhoun et al., 2008; Fu et al., 2008; Marquand et al., 2008; Cecchi et al., 2009; Arribas et al., 2010; Nouretdinov et al., 2011; Shen et al., 2010; Costafreda et al., 2011; Fan et al., 2011). Such fMRI-based diagnosis has the potential to assist psychiatrists in providing improved diagnosis and treatment for psychiatric patients. This approach is consistent with the recent emphasis on personalized medicine in health care delivery.

Resting state fMRI is attractive for fMRI-based diagnostics. For a resting state fMRI scan, the participant simply rests quietly without experiencing any stimulus presentation or performing an overt task (Raichle et al., 2001; Greicius et al., 2003; Fox et al., 2005). “Functional connectivity” analyses then allow one to examine the relationships of intrinsic activity patterns in various brain regions (see McIntosh and Gonzalez-Lima, 1994; Calhoun et al., 2001; Friston et al., 2003). Resting state fMRI could be deployed on existing clinical scanners without the need for MR-compatible task presentation hardware. It is also possible to acquire resting state fMRI scans with patient groups who have difficulty performing more complex psychological tasks. Differences between ADHD and control participants have previously been demonstrated with resting state fMRI (Cao et al., 2006, 2009; Tian et al., 2006, 2008; Zang et al., 2007; Castellanos et al., 2008; Uddin et al., 2008; Wang et al., 2009; Fair et al., 2010; Liston et al., 2011; Qiu et al., 2011; Sun et al., 2012). Resting state fMRI has also been used successfully to diagnose ADHD (Zhu et al., 2005, 2008) as well as schizophrenia (Shen et al., 2010). However, these studies included relatively small numbers of participants—20 in and 52 in Shen et al. (2010). The ADHD-200 dataset described in the next paragraph presented an important opportunity to test diagnosis based on resting state fMRI data with much larger participant numbers.

In summer 2011, the ADHD-200 Global Competition challenged teams to provide the best procedure for diagnosing individuals with ADHD from their resting state fMRI scans. Specifically, the goal was to assign each individual to one of three categories: ADHD-C, ADHD-I, or healthy control. (The ADHD-200 dataset contained only 11 individuals diagnosed with ADHD-H, and the ADHD-H category was therefore not considered in the diagnosis task.) The ADHD-200 Consortium made available a large Training Dataset that contained data from 776 participants collected at multiple institutions. Each participant's data included a resting state fMRI scan, a structural MRI scan, and individual characteristics data (age, gender, handedness, and IQ scores). The test (holdout) dataset was comprised of data from 197 additional participants for whom diagnostic information was not provided. Competition entrants had to build and train a diagnostic procedure using the 776 training participants and then submit predicted diagnostic labels for the 197 test set participants. Teams were ranked based on the accuracies of their predicted diagnostic labels. The ADHD-200 dataset is the first publicly-available dataset with fMRI scans from on-the-order-of one thousand participants, including both psychiatric patients and healthy controls. In contrast, previous studies on fMRI-based diagnosis (Zhu et al., 2005, 2008; Shinkareva et al., 2006; Calhoun et al., 2008; Fu et al., 2008; Marquand et al., 2008; Cecchi et al., 2009; Arribas et al., 2010; Nouretdinov et al., 2011; Shen et al., 2010; Costafreda et al., 2011; Fan et al., 2011) included 20–104 participants (median 39 across all 12 studies). The ADHD-200 dataset represents considerable effort and time on the part of its contributors and the ADHD-200 Global Competition organizers. As such, the competition provided an important and thus far unique opportunity to test fMRI-based diagnosis with a very large group of psychiatric patients and controls.

We compared the effectiveness of diagnosis based on participants' personal characteristic data with diagnosis based on participants' resting state fMRI scans. For fMRI-based diagnosis, we used a variety of feature extraction approaches including principal components analysis (PCA), the Fourier transform, and the functional connectivity (FC) analysis based on independent components analysis (ICA) of Calhoun et al. (2001) and Erhardt et al. (2011). A traditional group comparison was also done to identify differences in FC between ADHD patients and controls. We tested whether group differences in fMRI-derived features translated into differences among individual participants that could be used for accurate diagnosis. This paper will highlight the challenges of fMRI-based diagnosis posed by different sources of variance in large participant groups. We will also discuss group comparisons in contrast to individual differences analyses in terms of their diagnostic utility.

## 2. Materials and methods

### 2.1. Datasets

Data analyzed in this paper came from the ADHD-200 dataset, comprising data from 973 participants (for more details, see ADHD-200-Webpage, 2011). Each of the 973 participants was scanned at one of eight different sites, which pooled their data to make the ADHD-200 dataset. The eight sites were Peking University (PekingU), Bradley Hospital/Brown University (BrownU), Kennedy Krieger Institute (KKI), NeuroIMAGE Sample (NeuroIMAGE), New York University Child Study Center (NYU), Oregon Health and Science University (OHSU), University of Pittsburgh (UPitt), and Washington University in St. Louis (WashU); see ADHD-200-Webpage (2011). For every participant, at least one resting state fMRI scan was provided as well as a T1-weighted structural scan and several personal characteristic data points (age, gender, handedness, and for most participants one or more IQ scores). For the 2011 ADHD-200 Global Competition, the data were divided into two datasets. The ADHD-200 Training Dataset included 776 participants, and the holdout set (Test Release dataset) included 197 participants. For the competition, participants' diagnostic labels—healthy control, ADHD-C type, or ADHD-I type—as well as medication status and scores on various ADHD assessment instruments were given for the training set but not the holdout set. Competition entrants used the training set to train their diagnostic algorithms. They then applied those algorithms to the holdout set to generate predicted diagnoses for the 197 holdout participants, and they submitted these predicted diagnoses to the Competition organizers.

For all experiments on diagnosis using fMRI data as input, we used the subset of the ADHD-200 Global Competition Training Dataset derived by excluding the 108 participants whose resting state fMRI scans were given “questionable” quality assurance (QA) scores by the data curators. For the work described here, we refer to this dataset as the “Training Dataset.” (We refer to the original 776 participant ADHD-200 Training Dataset as the “Original Training Dataset.” Note that this is a superset of the Training Dataset). Our Training Dataset included 668 participants, whose details are shown in Table 1 below. Note that data from Brown University were included in the ADHD-200 Competition hold out set but not in the ADHD-200 Competition training set.

**Table 1:** **Details of Training Dataset participants**.

**Training set**	***n***	**Age (years)**	**Gender (%)**		**Handedness (%)**				**Medication history (%)**		
**Group**			**F**	**M**	**Left**	**Right**	**Ambi.**	**No data**	**None**	**Medicated**	**No data**
Control	429	12.4 ± 3.3	47.6	52.4	2.6	96.3	0.2	0.9	67.4	2.1	30.5
ADHD-C	141	11.4 ± 3.1	17.0	82.3	3.5	95.0	0.0	1.4	34.0	26.2	39.7
ADHD-I	98	12.1 ± 2.5	26.5	73.5	2.0	98.0	0.0	0.0	60.2	22.5	17.4
**Training set**	**Proportions by site (%)**										
**Group**	**PekingU**		**BrownU**	**KKI**		**NeuroIMAGE**	**NYU**		**OHSU**	**UPitt**	**WashU**
Control	27.0		0.0	13.5		5.1	21.2		8.4	15.4	9.3
ADHD-C	20.6		0.0	10.6		11.3	45.4		12.1	0.0	0.0
ADHD-I	50.0		0.0	5.1		0.0	33.7		11.2	0.0	0.0
**Training set**	**IQ scores (mean ± standard deviation)**										
**Group**	**Verbal IQ**			**Performance IQ**				**Full 2 IQ**		**Full 4 IQ**	
Control	114.9 ± 13.6			110.7 ± 13.5				112.2 ± 8.3		114.3 ± 13.3	
ADHD-C	110.2 ± 16.1			103.3 ± 14.0				NA		107.5 ± 13.9	
ADHD-I	106.9 ± 15.2			100.6 ± 15.3				NA		104.2 ± 14.1	

See section 2.1 for details and abbreviations.

IQ scores were provided for most Original Training Dataset participants except for those from NeuroIMAGE. (See Table 1 for IQ data summary.) KKI used the Wechsler Intelligence Scale for Children, Fourth Edition (WISC-IV). NYU, OHSU, and UPitt used the Wechsler Abbreviated Scale of Intelligence (WASI). WashU used two subtests of the WASI. PekingU used the Wechsler Intelligence Scale for Chinese Children-Revised (WISCC-R). NeuroIMAGE did not provide IQ scores for Training Dataset participants, and no Training Dataset participants came from BrownU. For each participant, from one to five IQ-related data points were provided. IQ Measure was provided for every Training Dataset participant and indicated which of the four above-listed IQ instruments was used or whether IQ data were absent for a given participant. The other four data points were Verbal IQ, Performance IQ, Full 2 IQ, and Full 4 IQ. Full 2 IQ and FULL 4 IQ were different estimations of the full scale IQ score. PekingU, KKI, NYU, and UPitt provided Verbal IQ, Performance IQ, and Full 4 IQ scores for their participants. UPitt also provided Full 2 IQ scores. OHSU and WashU provided Full 4 IQ scores.

Subsequent to the competition, diagnostic and medication data were released for the 197 ADHD-200 Holdout Dataset (“Test Release”) participants, except for the 26 participants from the BrownU site. In some analyses performed after the competition, we used the subset of the ADHD-200 Holdout Dataset consisting of the 171 participants for whom diagnostic data were released. We refer to this subset as the “Holdout Dataset.” (We refer to the original 197 participant holdout set as the “Original Holdout Dataset.”) For details of Holdout Dataset participants, see Table 2 below.

**Table 2:** **Details of Holdout Dataset participants**.

**Holdout set**	***n***	**Age (years)**	**Gender (%)**		**Handedness (%)**			**Medication history (%)**		
**Group**			**F**	**M**	**Left**	**Right**	**No data**	**None**	**Medicated**	**No data**
Control	94	12.0 ± 4.2	51.1	48.9	5.3	93.6	1.1	34.0	0.0	63.3
ADHD-C	51	11.5 ± 3.5	15.7	84.3	5.9	92.2	2.0	3.9	15.7	80.4
ADHD-I	26	12.1 ± 2.6	34.6	65.4	0.0	100.0	0.0	46.2	23.1	30.8
**Holdout set**	**Proportions by site (%)**							
**Group**	**PekingU**	**BrownU**		**KKI**	**NeuroIMAGE**		**NYU**	**OHSU**	**UPitt**	**WashU**
Control	28.7	0.0		8.5	14.9		12.8	29.8	5.3	0.0
ADHD-C	19.6	0.0		5.9	21.6		43.1	9.8	0.0	0.0
ADHD-I	53.8	0.0		0.0	0.0		26.9	3.8	15.4	0.0
**Holdout set**	**IQ scores (mean ± standard deviation)**									
**Group**	**Verbal IQ**			**Performance IQ**		**Full 2 IQ**			**Full 4 IQ**	
Control	119.2 ± 12.8			108.5 ± 13.6		100.6 ± 14.4			114.6 ± 12.3	
ADHD-C	109.2 ± 13.6			101.1 ± 17.0		93.3 ± 16.5			107.0 ± 14.8	
ADHD-I	108.6 ± 13.1			101.5 ± 11.2		NA			106.1 ± 11.2	

See section 2.1 for details and abbreviations.

IQ scores were provided for the Original Holdout Dataset participants. (See Table 2 for IQ data summary.) IQ testing details are identical to those for the Original Training Dataset (described above) with two exceptions. IQ scores were provided for NeuroIMAGE holdout participants (but not NeuroIMAGE Training Dataset participants). For Original Holdout Dataset participants, the NeuroIMAGE site used the Block Design and Vocabulary subtests of the WISC or Wechsler Adult Intelligence Scale (WAIS), depending on participant age. The use of this testing regimen was reflected in the IQ Measure (indicator) data point value for each NeuroIMAGE holdout set participant. A Full 2 IQ score was provided for all but one NeuroIMAGE holdout set participants. BrownU provided Verbal IQ, Performance IQ, and Full 4 IQ scores for its 26 participants. BrownU did not provide IQ Measure indicator data for its participants, nor do we have details of which IQ tests were used at BrownU.

Each data collection site used its own scanner(s) and its own MR scanning parameters. Full details are available at ADHD-200-Webpage (2011).

### 2.2. General diagnosis procedure

The ADHD-200 Global Competition required three-way diagnostic classification, but we also explored binary diagnosis because we suspected that it might be easier than three-way diagnosis. In binary diagnosis, we classified participants as healthy control vs. ADHD, collapsing across ADHD subtype. In three-way diagnosis, we classified participants as healthy control vs. ADHD-C vs. ADHD-I.

Depending on the analysis, we used as input to the diagnosis process either personal characteristic data or fMRI data. We first preprocessed the data, applied dimensionality reduction algorithms in the case of fMRI data, and then extracted a feature vector for each participant. Personal characteristic feature vectors included site of data collection, age, gender, handedness, and IQ scores (for details see section 2.3). We tested four different types of fMRI feature vector: mean fMRI signal intensity over time in each voxel (spatial location), projections of voxels' timecourses into a PCA space, low frequency Fourier components of voxel's timecourses, and voxels' weightings on FC maps derived from ICA. (For details of preprocessing and feature extraction, see sections 2.4 and 2.5.) Participant feature vectors and diagnostic labels were input into the Weka machine learning software package (Hall et al., 2009). We used Weka's implementations of the logistic classifier, linear support vector machine (SVM), quadratic SVM, cubic SVM, and radial basis function (RBF) SVM classifiers. Each classifier had free parameters that were fit to the data during training. The trained classifier could then predict a participant's diagnostic category from his or her feature vector.

We will refer to a given combination of input data choice (personal characteristic vs. fMRI data), preprocessing, feature extraction, and learning algorithm as a “Diagnostic Pipeline.” To test the binary and three-way diagnostic performance of various Diagnostic Pipelines, we used 10-fold cross validation. That is, we defined a standard set of 10-folds where each fold included a (disjoint) subset of 66 or 67 participants from the 668 Training Dataset participants. The subsets were approximately counterbalanced for diagnostic class, gender, age, handedness, IQ, medication status, and site of data collection. (Perfect counterbalancing was not possible due to the make-up of the Training Dataset.) For each iteration *i* of 10-fold cross validation, we trained a classifier on the feature vectors from participants in all folds except fold *i*. The test set accuracy score for a given fold *i* was defined as the proportion of participants in fold *i* assigned to the correct diagnostic category by the classifier. The classifier was not given any of the test set data during training, so testing on the test set data gave an indication of the classifier's ability to generalize diagnostic performance to new participants' data. To derive a training set accuracy score, we also tested the trained classifier on the same data used for training—namely all participants except for those in fold *i*. The training set accuracy provided a measure of the classifier's ability to detect some diagnostic pattern in the data. Poor performance on the training set indicated either that the data in question did not contain diagnostically-useful information or that the classifier was not able to detect diagnostically-useful information that was present. The mean and standard deviation were computed for the 10 test set accuracy scores and for the 10 training set accuracy scores for each Diagnostic Pipeline. We compared different Diagnostic Pipelines' accuracies from 10-fold cross validation using one-tailed, paired samples *t*-tests (*df* = 9). We also compared accuracy results to the baseline chance accuracy achieved by guessing healthy control (i.e., the majority class) for every participant. Note that this definition of chance accuracy is different from the one used by the ADHD-200 Global Competition organizers (see ADHD-200-Results-Webpage, 2011).

For the ADHD-200 Global Competition, we tested various Diagnostic Pipelines on the three-way diagnosis task using 10-fold cross validation. We selected the best-performing Diagnostic Pipeline, which turned out to use the logistic classifier with only personal characteristic data as input. Since fMRI data quality did not affect personal characteristic data, we trained the logistic classifier on all 776 participants from the Original Training Dataset. We applied this trained classifier to the 197 ADHD-200 Global Competition Original Holdout Dataset participants to generate predicted diagnostic labels.

In follow-up analyses after the competition, we tested some other Diagnostic Pipelines (see below) using 10-fold cross validation. Testing dozens of different Diagnostic Pipelines introduced the problem of multiple comparisons and over-fitting. To address this issue, we selected the best-performing Diagnostic Pipeline in a given context (e.g., binary diagnosis using only personal characteristic data as input) and trained its classifier on all 668 participants from the Training Dataset. We then tested the trained classifier on the 171 participants from our Holdout Dataset. For these analyses, the Holdout Dataset was not used in any way to select a Diagnostic Pipeline nor to train its classifier. The Holdout Dataset was used only to test the ability of a given trained Diagnostic Pipeline to generalize its performance to completely new data.

### 2.3. Diagnosis with personal characteristic data

We tested diagnostic classification using just personal characteristic data as input with no MR imaging data. Personal characteristic data were preprocessed in two steps. The selection process selected a subset of personal characteristic data features to include. Personal characteristic selection 1 (*PCs1*) included 7 features: data collection site, gender, age, handedness, Verbal IQ, Performance IQ, and Full 4 IQ. Personal characteristic selection 2 (*PCs2*) was identical to *PCs1* except for the addition of two more features (IQ Measure and Full 2 IQ) as well as different preprocessing of handedness data from the NeuroIMAGE site. *PCs2* included nine features: data collection site, gender, age, handedness, IQ Measure, Verbal IQ, Performance IQ, Full 2 IQ, and Full 4 IQ. Handedness data from all sites except the NeuroIMAGE site were categorical (0 = left-handed, 1 = right-handed, 2 = ambidextrous). NeuroIMAGE handedness values were continuous scores from the Edinburgh Handedness Inventory (Oldfield, 1971). *PCs1* simply took all handedness scores as they were and treated handedness as a continuous feature. *PCs2* modified the NeuroIMAGE handedness scores to fit the 0, 1, 2 categorical scheme by replacing all positive-valued Edinburgh Handedness scores with a 1 (right-handed) and all negative scores with a 0 (left-handed). The second preprocessing step filled in missing values using mean imputation for continuous (real-valued) features and mode imputation for categorical features. After being imported into the Weka machine learning package, feature vectors were standardized such that each feature element fell in the range 0–1 [see section G as well as Witten et al. (2011)]. We used *PCs1* in tests prior to the ADHD-200 Global Competition deadline. Our competition submissions were also generated using *PCs1*. We used both *PCs1* and *PCs2* in follow-up analyses after the competition.

### 2.4. fMRI data preprocessing

**Figure 1:**
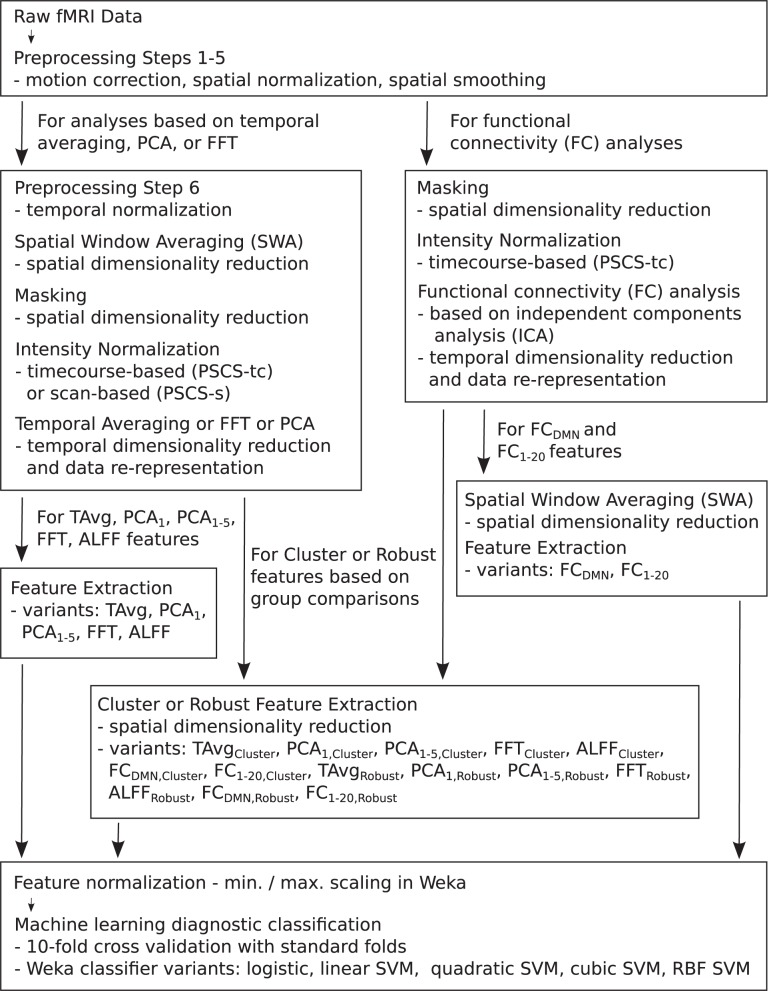
**Flow chart summarizing fMRI data preprocessing, normalization, dimensionality reduction, feature extraction, and testing in diagnosis tasks**.

For the resting state fMRI data, we used our own preprocessing pipeline based on the SPM8 fMRI analysis package as well as in-house MATLAB code, as opposed to the preprocessed data supplied by the ADHD-200 Global Competition organizers. Preprocessing for each participant included: (1) 6 parameter rigid body motion correction in SPM8, (2) rigid body co-registration of functional scans to subject-specific anatomical scans in SPM8, (3) non-linear spatial warping (estimation and interpolation) of each subject's anatomical volume to the MNI T1 template space at 1 × 1 × 1 mm resolution in SPM8, (4) interpolation of fMRI volumes into the T1 template space at 3 × 3 × 3 mm spatial resolution using warping parameters from step (3), (5) 8 mm full width at half maximum (FWHM) Gaussian spatial filtering of fMRI volumes in SPM8. At this point, all participants' resting state fMRI data were aligned in the MNI T1 template space, and they had the same spatial dimensions (57 × 67 × 50 voxels) and the same spatial resolution (3 × 3 × 3 mm voxel size). The fMRI data still varied in temporal duration and sampling rate depending on the participant and site of data collection. Preprocessing step (6) involved truncation of all resting state fMRI data to length 185 s and temporal linear interpolation of all fMRI scans into a sampling rate of 2 Hz or 0.5 s volume time (see Appendix B for details and motivation of temporal preprocessing). After step (6), participant fMRI data had the same temporal dimensions (370 time points with a 0.5 s volume time). For the FC Analysis (end of section 2.5), we did not use preprocessing step (6) on the data. All other fMRI analyses included step (6). Also see Figure 1.

### 2.5. fMRI dimensionality reduction and feature extraction

Each preprocessed fMRI scan was very high dimensional and included 70,651,500 separate intensity values (57 × 67 × 50 spatial grid by 370 time points)—about 280 Mb using single floating point precision. In comparison to 70,651,500, the number of participants (668) in the Training Dataset was small. Therefore, a machine learning algorithm trained on the full fMRI dataset would very likely over-fit to high dimensional noise rather than learning general diagnostic patterns in the data. To address this problem, we used several different methods for reducing spatial and temporal dimensionality. Dimensionality reduction was also necessary to reduce computation times and memory loads to manageable levels during classifier training and testing. To create each Diagnostic Pipeline, we combined dimensionality reduction procedures as described below. (Also see summary in Figure 1 as well as mathematical details in the Appendices.)

Spatial window averaging (SWA) was similar to down-sampling (see Appendix D). It took a reduction parameter *r* and reduced the spatial dimensionality by a factor of approximately *r* along each spatial axis. We considered various values for *r*. *r* = 1 produced no dimensionality reduction. *r* = 3 reduced the original volume size from 57 × 67 × 50 (190,950) voxels to 19 × 22 × 16 (6,688) voxels. *r* = 8 reduced the original volume to 7 × 8 × 6 (336) voxels. For a 4D fMRI scan data array, SWA was applied separately to each 3D volume (time point).

We used a binary mask to remove voxels outside the brain (see Appendix E). Masking reduced the original 57 × 67 × 50 volume's voxel count from 190,950 to 97,216. For SWA-reduced data, masking reduced a 19 × 22 × 16 volume (produced with SWA, *r* = 3) from 6,688 voxels to 3,558. For a 7 × 8 × 6 volume (produced with SWA, *r* = 8), masking reduced the voxel count from 336 to 186.

fMRI scan intensities differed across participants and, in particular, across different data collection sites. We used two different intensity normalization methods. Timecourse-based intensity normalization involved percent signal change scaling of each voxel's timecourse (PSCS-tc). Scan-based intensity normalization involved percent signal change scaling of each fMRI scan (PSCS-s) such that each voxel's timecourse was scaled by the mean across the entire dataset (after SWA and masking). Also see Appendix C.

After SWA, masking, and intensity normalization, fMRI data were processed with one of four different temporal dimensionality reduction procedures based on temporal averaging (T Avg), PCA, the Fast Fourier Transform (FFT), or an FC analysis based on ICA.

T Avg reduced the temporal dimensionality of a 4D fMRI scan from 370 to 1 by replacing each voxel's timecourse with the average of that timecourse. This procedure was combined with SWA using *r* = 3 or *r* = 8 and binary masking. Only scan-based intensity normalization (PSCS-s) was used with temporal averaging. Timecourse-based normalization (PSCS-tc) was not used with T Avg as it set every voxel's mean intensity to zero, which would have resulted in all-zero feature vectors with T Avg. T Avg feature vectors had a length of 3558 for *r* = 3 or 186 for *r* = 8.

We used PCA implemented in MATLAB to reduce temporal dimensionality (see Appendix F). We treated the timecourse vector from each voxel from each participant as an observation and performed PCA on the *n*_participants_ × *n*_voxels_ timecourses. We kept the first 1 or first 5 principal components and projected each voxel's timecourse onto those components, for each participant. This resulted in a temporal size reduction from 370 to 1 or from 370 to 5. We will refer to these two cases as PCA_1_ and PCA_1−5_, respectively. For Diagnostic Pipelines incorporating PCA dimensionality reduction, we used timecourse- or scan-based intensity normalization (PSCS-tc or PSCS-s). We also used SWA (*r* = 8) and masking to reduce PCA computation time. PCA_1_ feature vectors had a length of 186, while PCA_1−5_ feature vectors had a length of 930.

We used the FFT in two different temporal dimensionality reduction procedures. In the FFT feature extraction procedure, we computed the FFT of each length 370 timecourse from each voxel from each participant and retained the modulus and phase angle from the 18 (complex-valued) FFT components in the 0.001–0.1 Hz frequency range. This frequency range was chosen based on the literature on analysis of low frequency fluctuations (ALFF) (Biswal et al., 1995; Zang et al., 2007). FFT reduced temporal dimensionality from 370 to 36. In the ALFF procedure, we computed the mean modulus over the 18 FFT components in the 0.001–0.1 Hz frequency range. ALFF reduced temporal dimensionality from 370 to 1. FFT and ALFF feature extraction were combined with SWA reduction (for FFT *r* = 8, for ALFF *r* = 3 or *r* = 8), masking, and timecourse- or scan-based intensity normalization (PSCS-tc or PSCS-s). FFT Diagnostic Pipelines produced feature vectors with length 6696. ALFF feature vectors had a length of 3558 for *r* = 3 or 186 for *r* = 8.

We performed an FC analysis on the fMRI data using spatial ICA (Calhoun et al., 2001; Erhardt et al., 2011). (Also see Figure 1.) For details of this FC analysis, see Calhoun et al. (2001) and Erhardt et al. (2011). Briefly, the FC analysis was as follows. fMRI data underwent preprocessing steps 1–5 (see section 2.4.) Each scan was masked. Voxel timecourses were normalized using PSCS-tc. We did not use percent signal change scaling based on the entire scan mean (PSCS-s) for FC analysis as PSCS-s does not normalize out between-voxel mean intensity differences as is required for this FC analysis. Each participant's data were entered into a separate spatial PCA, keeping the first 25 components. The 25 PCA components from each participant were concatenated and entered into a second PCA step, in which 20 components were retained. These 20 components underwent ICA using Hyvarinen's FastICA algorithm (Hyvarinen, 1999). We used Erhardt et al. (2011)'s GICA3 method for back reconstruction of individual participant's ICA components from the group components. This resulted in 20 volumetric FC weighting maps for each participant. (Note: In reconstructing the 3D volumetric maps, those voxels that had been previously masked out were simply zero-filled.) Each FC weighting map depicted either a network of brain regions or else a noise pattern (e.g., movement noise). We identified the default mode network (DMN, Raichle et al., 2001; Fox et al., 2005) from among the 20 FC weighting maps by visual inspection. The default mode or resting state network includes posterior cingulate cortex, precuneus, medial prefrontal cortex, and temporal-parietal cortex. We extracted feature vectors from the FC maps as components of Diagnostic Pipelines. FC_DMN_ feature extraction included SWA of the DMN map for a given participant with reduction factor *r* = 3 or *r* = 8, followed by masking and flattening (see Appendix A) to generate a feature vector. FC_DMN_ reduced the temporal dimensionality of the data from 370 to 1. With *r* = 3 and *r* = 8, respectively, FC_DMN_ Diagnostic Pipelines produced feature vectors with lengths 3558 and 186. FC_1−20_ feature extraction included SWA of all 20 FC weighting maps for a given participant with reduction factor *r* = 8 followed by masking, flattening, and concatenation of the 20 maps to create a feature vector for each participant. FC_1−20_ reduced the temporal dimensionality of the data from 370 to 20. With *r* = 8, FC_1−20_ Diagnostic Pipelines produced feature vectors of length 3720.

### 2.6. Diagnostics from group differences

We also tested whether group differences identified using statistical comparisons of ADHD vs. controls or ADHD-C vs. ADHD-I patients would produce features with good diagnostic properties. For each of the feature extraction methods discussed in section 2.5 (T Avg, PCA_1_, PCA_1−5_, FFT, ALFF, FC_DMN_, and FC_1−20_), we performed statistical group comparisons, either between ADHD patients and controls or between ADHD patient subtypes, and selected clusters of voxels that showed significant differences in these comparisons. Participants' feature vectors were then extracted from those clusters. Feature extraction procedures using this method are denoted T Avg_Cluster_, PCA_1,Cluster_, PCA_1−5,Cluster_, FFT_Cluster_, ALFF_Cluster_, FC_DMN,Cluster_, and FC_1−20,Cluster_.

In detail, the cluster identification procedure was as follows. Each of the feature extraction methods from section 2.5 produced a length η feature vector at each voxel location that passed masking. η took on the following values: T Avg:1, PCA_1_:1, PCA_1−5_:5, FFT:36, ALFF:1, FC_DMN_:1, and FC_1−20_:20. For example, in a given participant, PCA_1−5_ produced a length five feature vector at each voxel containing that voxel's weights for the first five PCA components. Consider a given feature extraction method (T Avg, PCA_1_, PCA_1−5_, FFT, ALFF, FC_DMN_, or FC_1−20_) and a given participant *p*. Let *k* ∈ [1, η] be an index into the length η feature vector. For PCA_1−5_, *k* = 1 denotes the first PCA component weight, *k* = 2 denotes the second, and so on. We define **V_k,p_** (where *k* ∈ [1, η]) as the 3D volume comprised of the *k*th feature value at each voxel. For PCA_1−5_, **V_1,p_** contained the weights for the first PCA component for participant *p*. (Voxels of **V_k,p_** that did not pass the masking process were simply zero-filled.) For each *k* ∈ [1, η], we identified clusters of voxels (regions of interest) exhibiting significant differences in two-sample *t*-tests between groups. Specifically, statistical contrast maps were generated with massively univariate *t*-tests performed at each voxel location on the values from participants' volumes **V_k,p_**. Diagnostic Pipelines included clusters either from the comparison of ADHD patients vs. healthy controls (*n*_comparisons_ = 1) or from the comparison of both ADHD patients vs. healthy controls as well as ADHD-C vs. ADHD-I patients (*n*_comparisons_ = 2). That is, a total of η × *n*_comparisons_ statistical maps were computed for a given Diagnostic Pipeline. Statistical contrast maps were thresholded voxelwise such that |*t*| > θ_*t*_, where the value of θ_*t*_ was specific to each Diagnostic Pipeline. Statistical maps were then cluster size thresholded (see Appendix I) to zero out those voxels belonging to clusters with volumes (in mm^3^) less than the threshold volume θ_cs_. The value for θ_cs_ was also specific to each Diagnostic Pipeline. Voxel clusters were extracted from the thresholded contrast maps using an automated algorithm (see Appendix H). To avoid over-fitting, on a given fold *i* of 10-fold cross validation, the statistical cluster selection was done on the training data only (participants not in fold *i*). The parameter *n*_clusters_ governed how many significant clusters were retained from each statistical map in a given Diagnostic Pipeline. Clusters were ordered by statistical mass, defined as the sum of absolute *t*-values for all voxels in a cluster. The *n*_clusters_ most statistically massive clusters from each statistical map were retained, with less massive clusters discarded. Setting *n*_clusters_ = ∞ resulted in all clusters' being kept. Finally, for a given participant *p*, for each cluster identified in each of the η × *n*_comparisons_ statistical maps, we took the appropriate **V_k,p_** and computed its mean value across the voxel locations in that cluster. All such mean values were then concatenated to form a feature vector for participant *p*.

The T Avg_Cluster_ feature extraction method was combined with no SWA spatial reduction (equivalent to SWA with *r* = 1), masking, and scan-based intensity normalization (PSCS-s). PCA_1,Cluster_ and PCA_1−5,Cluster_ feature extraction were combined with SWA (*r* = 8), masking, and either timecourse- or scan-based intensity normalization (PSCS-tc or PSCS-s). FFT_Cluster_ feature extraction was combined with SWA (*r* = 3), masking, and either PSCS-tc or PSCS-s intensity normalization. The ALFF_Cluster_ feature extraction method was combined with no SWA spatial reduction, masking, and either PSCS-tc or PSCS-s. FC_DMN,Cluster_ and FC_1−20,Cluster_ feature extraction were always combined with no SWA spatial reduction, masking, and timecourse-based intensity normalization (PSCS-tc). The T Avg_Cluster_, PCA_1,Cluster_, PCA_1−5,Cluster_, FFT_Cluster_, ALFF_Cluster_, FC_DMN,Cluster_, and FC_1−20,Cluster_ procedures were governed by five parameters, including θ_*t*_, θ_cs_, and *n*_clusters_ defined above, as well as the minimum inter-peak distance *d*_min_ and minimum extracted cluster size *v*_min_ parameters used by the automated cluster extractor (see Appendix H). We tested Diagnostic Pipelines with different settings for these parameters. Let us consider the parameter vector [θ_*t*_, θ_cs_, *n*_clusters_, *d*_min_, *v*_min_]. This parameter vector took on a specific value for each Diagnostic Pipeline. For T Avg_Cluster_, we tested the parameter vector value [2.582, 1701, ∞, 30, 540]. For PCA_1,Cluster_ and PCA_1−5,Cluster_, we tested the parameter vectors [2.582, 0, ∞, 0, 0] and [1.964, 0, ∞, 0, 0]. For FFT_Cluster_, we tested the parameter vector [2.582, 1458, ∞, 27, 0]. For ALFF_Cluster_, we tested the parameter vector [2.582, 1701, ∞, 30, 540]. For FC_DMN,Cluster_, we tested the following parameter vectors: [2, 1350, 10, 30, 540], [2.582, 1701, 5, 30, 540], and [2.582, 1701, ∞, 30, 540]. For FC_1−20,Cluster_, we tested the parameter vector [2, 1350, 10, 30, 540].

We tested a variant of the cluster-based feature extraction method described above. This variant was intended to reduce cluster variability across the different iterations of 10-fold cross validation. Feature extraction methods utilizing this variant were denoted T Avg_Robust_, PCA_1,Robust_, PCA_1−5,Robust_, FFT_Robust_, ALFF_Robust_, FC_DMN,Robust_, and FC_1−20,Robust_. For a given iteration *i* of 10-fold cross validation, a set *S*_*i*_ of statistical comparison maps was computed from all participants not in fold *i*. The set *S*_*i*_ included η × *n*_comparisons_ statistical comparisons on the volumes **V_k,p_** where *k* ∈ [1, η] and *p* ∈ [1, *n*_participants_] (see above for definitions). For example, in FC_DMN,Robust_, *S*_*i*_ included statistical comparisons on the DMN FC weighting map, as in FC_DMN,Cluster_. For FC_1−20,Robust_, *S*_*i*_ included statistical comparisons on all 20 FC weighting maps, as in FC_1−20,Cluster_. Then, we also computed nine additional sets of comparisons maps *S*_*i*, *j*_ in nine sub-iterations *j*, where *j* ∈ {1, …, 10}\{*i*}. A given *S*_*i*, *j*_ was equivalent to a given *S*_*i*_ except that *S*_*i*, *j*_ was computed from participants not in fold *i* nor in fold *j*. The *S*_*i*, *j*_ maps underwent local |*t*|-value thresholding as well as cluster size thresholding (see Appendix I). Here, we retained only those voxel locations in *S*_*i*_ that also exhibited significance on all nine sets *S*_*i*, *j*_. That is, after all nine sub-iterations *j*, the maps in *S*_*i*_ were modified by zeroing out those voxel locations not showing significance in the appropriate maps from the sets *S*_*i*, *j*_. Clusters were then extracted from the filtered maps *S*_*i*_ and feature vectors generated for each participant as in the cluster-based feature extraction method described above. This somewhat elaborate process was meant to introduce robustness into the cluster selection process, as it only considered voxel locations showing consistent statistical differences across different subsets of participants.

The T Avg_Robust_, PCA_1,Robust_, PCA_1−5,Robust_, FFT_Robust_, ALFF_Robust_, FC_DMN,Robust_, and FC_1−20,Robust_ feature extraction methods were combined with preprocessing steps in the same way, respectively, as the T Avg_Cluster_, PCA_1,Cluster_, PCA_1−5,Cluster_, FFT_Cluster_, ALFF_Cluster_, FC_DMN_,Cluster, and FC_1−20,Cluster_ methods. (See above for details.) The robust feature extraction methods were controlled by the same vector of parameters [θ_*t*_, θ_cs_, *n*_clusters_, *d*_min_, *v*_min_] introduced above for the cluster-based methods. For T Avg_Robust_, we tested the parameter vector value [2.582, 1701, ∞, 30, 540]. For PCA_1,Robust_ and PCA_1−5,Robust_, we tested the parameter vector [1.964, 0, ∞, 0, 0]. For FFT_Robust_, we tested the parameter vector [2.582, 1458, ∞, 27, 0]. For ALFF_Robust_, we tested the parameter vector [2.582, 1701, ∞, 30, 540]. For FC_DMN_,Robust, we tested the parameter vector [2.582, 63, ∞, 0, 0]. For FC_1−20,Robust_, we tested the following values for the parameter vector: [2.582, 63, ∞, 0, 0], [2.6, 100, ∞, 0, 0], [2.6, 50, ∞, 0, 0], [2.6, 0, ∞, 0, 0], [4, 0, ∞, 0, 0], and [2.6, 200, ∞, 600, 5400].

### 2.7. fMRI functional connectivity analysis group comparison

We performed statistical group comparison analyses on the FC weighting maps (see last paragraph of section 2.5). These comparisons took the form of massively univariate *t*-tests on the weighting values for each voxel location for each map. We considered two different *t*-tests. The localizer test compared all participants' weighting values against zero. The patients vs. controls *t*-test contrasted ADHD participants' FC weighting maps (collapsing across ADHD subtype) against controls. The resulting statistical maps were thresholded voxel-wise at |*t*|≥2.582 (*p* < 0.01, two-tailed *t*-test, *df* = 666). To correct for multiple comparisons across the voxel population at a global *p* < 0.05, all maps were cluster size thresholded (see Appendix I) with a minimum cluster size of 63 voxels (equivalent to 1701 mm^3^). The cluster size threshold of 63 was determined using Monte Carlo simulation.

## 3. Results

### 3.1. Diagnosis with personal characteristic data

Table 3 shows accuracy scores achieved using personal characteristic data features for the binary and three-way diagnostic tasks. We defined chance baseline as the accuracy obtained from guessing the majority class (healthy control) for all participants. Chance accuracy was 64.2% for the Training Dataset. The logistic classifier, linear SVM, quadratic SVM, and cubic SVM classifiers all performed significantly better than chance on both binary and three-way diagnosis (see Table 3) using either the personal characteristic data selection 1 (*PCs1*) or selection 2 (*PCs2*) features (see section 2.3). The RBF SVM classifier always guessed healthy control. The best binary diagnostic accuracy (75.0 ± 4.5%) was better than the best three-way diagnostic accuracy (69.0 ± 8.3) at *p* = 0.01 (two-tailed paired samples *t* = 3.24, *df* = 9).

**Table 3:** **Training Dataset results: accuracies for binary and three-way diagnosis using various classifiers with personal characteristic data from the 668 participant Training Dataset**.

**Diagnostic task**	**Input data**	**Classifier**	**Accuracy (%)**	***P* value**
Binary	Chance		64.2 ± 3.8
	Personal characteristic	Logistic	73.7 ± 5.1	1 × 10^−5^
	data selection 1 (*PCs1*)	Linear SVM	74.4 ± 4.6	9 × 10^−6^
		Quadratic SVM	**74.5** ± **6.2**	4 × 10^−5^
		Cubic SVM	74.4 ± 4.8	2 × 10^−6^
		RBF SVM	64.2 ± 3.8	NA
	Personal characteristic	Logistic	74.0 ± 5.0	1 × 10^−5^
	data selection 2 (*PCs2*)	Linear SVM	**75.0** ± **4.5**	7 × 10^−6^
		Quadratic SVM	74.2 ± 6.3	5 × 10^−5^
		Cubic SVM	73.8 ± 5.0	3 × 10^−5^
		RBF SVM	64.2 ± 3.8	NA
Three-way	Chance		64.2 ± 3.8
	Personal characteristic	Logistic	**68.7** ± **8.1**	0.020
	data selection 1 (*PCs1*)	Linear SVM	66.9 ± 7.1	0.038
		Quadratic SVM	68.6 ± 7.5	0.006
		Cubic SVM	68.6 ± 8.2	0.020
		RBF SVM	64.2 ± 3.8	NA
	Personal characteristic	Logistic	**69.0** ± **8.3**	0.020
	data selection 2 (*PCs2*)	Linear SVM	66.9 ± 7.2	0.050
		Quadratic SVM	68.9 ± 7.9	0.009
		Cubic SVM	67.5 ± 7.7	0.046
		RBF SVM	64.2 ± 3.8	NA

PCs1, personal characteristic data selection 1; *PCs2*, personal characteristic data selection 2. See section 2.3 for details. Accuracy column shows mean accuracies ± standard deviations across 10-fold cross validation. Best result in a given category is shown in bold font. *P* value column shows *p* values for one-tailed, paired samples *t*-tests (*df* = *9*) of accuracies against chance baseline (guessing control for every participant). NA (not available) indicates that the *t*-test was undefined in the case of SVM (RBF), which always guessed control and produced predictions identical to chance baseline.

At the time of the ADHD-200 Global Competition deadline, the logistic classifier using personal characteristic data selection 1 (*PCs1*) provided the best accuracy on three-way diagnosis of any Diagnostic Pipeline we had tested, including the fMRI-based Diagnostic Pipelines discussed below. (Recall that “Diagnostic Pipeline” refers to the combination of input data, preprocessing, feature extraction, and learning algorithm used to create a predictor that can produce a diagnosis for each participant.) The logistic classifier with *PCs2* input achieved slightly better accuracy than with *PCs1* on the Training Dataset, but we did not test *PCs2* until after the competition. Therefore, we used the logistic classifier with *PCs1* input features trained on the entire ADHD-200 Original Training Dataset to generate our predicted diagnostic submissions for the competition based on the ADHD-200 Original Holdout Dataset. Our submission achieved the highest prediction accuracy (62.52%) as well as the best competition score (124 out of 195) of any of the 21 competing teams. The competition scoring system assigned one point for every correct diagnosis and 0.5 point for a diagnosis of ADHD but with the wrong subtype (e.g., diagnosing ADHD-I when the participant was actually in the ADHD-C category). For more details of competition results, see ADHD-200-Results-Webpage (2011).

We tested the four best-performing Diagnostic Pipelines from Table 3 on the 171 participant Holdout Dataset, for which diagnostic labels were released after the ADHD-200 Global Competition. The results of these holdout tests are shown in Table 4. All four Diagnostic Pipelines performed better than chance, indicating their ability to generalize to new data that was not used either to train the classifier nor to select the learning algorithms.

**Table 4:** **Holdout Dataset results: accuracies for binary and three-way diagnosis on the 171 participant Holdout Dataset using personal characteristic input data**.

**Diagnostic task**	**Input data**	**Classifier**	**Holdout accuracy (%)**
Binary	Chance		55.0
	*PCs*1	Quadratic SVM	69.0
	*PCs*2	Linear SVM	65.5
Three-way	Chance		55.0
	*PCs*1	Logistic	63.7
	*PCs*2	Logistic	59.1

Only the four best-performing Diagnostic Pipelines from Table 3 were tested on the Holdout Dataset. *PCs1*, personal characteristic data selection 1; *PCs2*, personal characteristic data selection 2. See section 2.3 for details. Holdout accuracy column shows accuracies derived from training each classifier on the 668 participant Training Dataset and then testing it on the Holdout Dataset. Chance baseline was derived by guessing control for every participant.

### 3.2. Diagnosis with fMRI data

**Figure 2:**
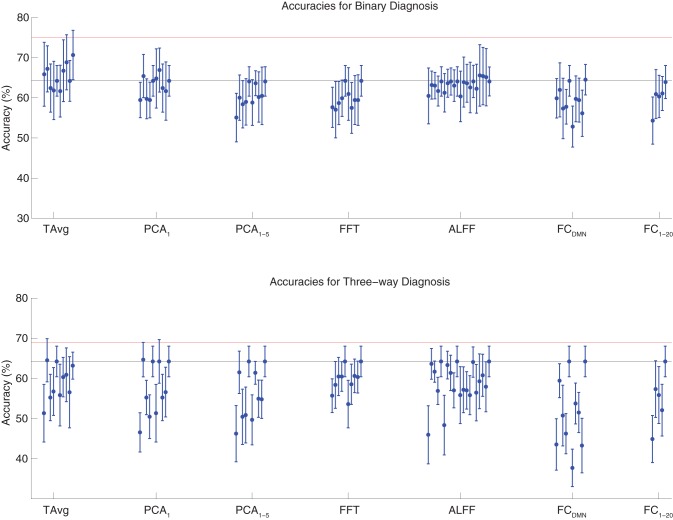
**Top**: Accuracies on the binary diagnosis task (controls vs. ADHD) using fMRI input data after dimensionality reduction/feature extraction with the T Avg, PCA_1_, PCA_1−5_, FFT, ALFF, FC_DMN_, or FC_1−20_ procedures (section 2.5) with five different classifiers (section 2.2). Each blue dot with error bars shows the mean accuracy and standard deviation for one Diagnostic Pipeline (feature data and classifier combination) on 10-fold cross validation with the 668 participant Training Dataset. The black horizontal line at 64.2% accuracy indicates chance baseline (guessing healthy control for all participants). The red horizontal line at 75.0% accuracy indicates the best mean accuracy achieved with binary diagnosis on the Training Dataset using personal characteristic input data (section 3.1). **Bottom**: Accuracies on the three-way diagnosis task (controls vs. ADHD-C vs. ADHD-I) using same Diagnostic Pipelines as in **top panel**. The red horizontal line at 69.0% accuracy indicates the best mean accuracy achieved with three-way diagnosis on the Training Dataset using personal characteristic input data (section 3.1). Other conventions as in **top panel**.

We tested binary diagnosis (Figure 2 top) and three-way diagnosis (Figure 2 bottom) using resting state fMRI input data with T Avg, PCA_1_, PCA_1−5_, FFT, ALFF, FC_DMN_, and FC_1−20_ feature extraction (see section 2.5). None of these fMRI-based features provided better diagnostic accuracy than the best results using personal characteristic data (section 3.1) on either diagnostic task.

The best accuracy on binary diagnosis of the tests shown in Figure 2 was 70.7 ± 6.2%, which was achieved by the Diagnostic Pipeline that included SWA with reduction factor *r* = 3, scan-based percent signal change intensity normalization (PSCS-s), T Avg, and the RBF SVM classifier. This accuracy was significantly better than the 64.2 ± 3.8% chance accuracy (*p* = 0.008, *t* = 3.00, *df* = 9). However, this accuracy was worse than the 75.0 ± 4.5% obtained with the best performing Diagnostic Pipeline using personal characteristic data (section 3.1) at *p* = 0.04 (*t* = 1.94, *df* = 9).

The best accuracy on three-way diagnosis of the tests shown in Figure 2 was 64.7 ± 4.3%. The Diagnostic Pipeline that obtained this accuracy used SWA with reduction factor *r* = 8, timecourse-based percent signal change intensity normalization (PSCS-tc), PCA_1_ feature extraction, and the linear SVM classifier. Though this accuracy was barely above the baseline chance accuracy of 64.2 ± 3.8%, this small difference was significant (*p* = 0.04, *t* = 1.96, *df* = 9). This accuracy was worse than the 69.0 ± 8.3% obtained with the best performing Diagnostic Pipeline using personal characteristic data (section 3.1) at *p* = 0.03 (*t* = 2.23, *df* = 9).

### 3.3. Diagnosis with fMRI data using cluster and robust feature extraction

**Figure 3:**
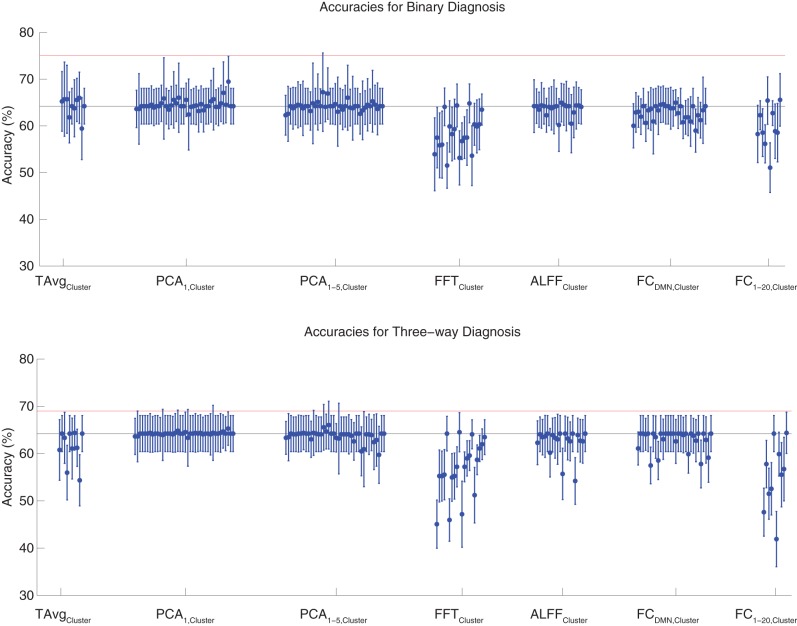
**Top**: Accuracies on the binary diagnosis task (controls vs. ADHD) using features derived from clusters of significant difference on group comparisons (section 2.6) with five different classifiers (section 2.2). Each blue dot with error bars shows the mean accuracy and standard deviation for one Diagnostic Pipeline (feature data and classifier combination) on 10-fold cross validation with the 668 participant Training Dataset. The black horizontal line at 64.2% accuracy indicates chance baseline (guessing healthy control for all participants). The red horizontal line at 75.0% accuracy indicates the best mean accuracy achieved with binary diagnosis on the Training Dataset using personal characteristic input data (section 3.1). **Bottom**: Accuracies on the three-way diagnosis task (controls vs. ADHD-C vs. ADHD-I) using same Diagnostic Pipelines as in **top panel**. The red horizontal line at 69.0% accuracy indicates the best mean accuracy achieved with three-way diagnosis on the Training Dataset using personal characteristic input data (section 3.1). Other conventions as in **top panel**.

Binary and three-way diagnosis were tested with feature extraction methods based on significant clusters identified in group comparisons. These feature extraction methods included the T Avg_Cluster_, PCA_1,Cluster_, PCA_1−5,Cluster_, FFT_Cluster_, ALFF_Cluster_, FC_DMN,Cluster_, and FC_1−20,Cluster_ procedures (see section 2.6). Results are shown in Figure 3. None of these tests performed better than the best Diagnostic Pipelines using personal characteristic data (section 3.1).

For binary diagnosis, the best cluster-based Diagnostic Pipeline achieved 69.5 ± 5.5% accuracy, which was significantly better than the baseline chance accuracy of 64.2 ± 3.8% (*p* = 0.002, *t* = 3.88, *df* = 9). This Diagnostic Pipeline used SWA with reduction factor *r* = 8, scan-based percent signal change intensity normalization (PSCS-s), PCA_1,Cluster_ feature extraction based on comparison of ADHD patients vs. controls as well as ADHD-C vs. ADHD-I patients, and the cubic SVM classifier. In comparison to this cluster-based approach, the best personal characteristic-based Diagnostic Pipeline (section 3.1) achieved a significantly better accuracy of 75.0 ± 4.5% (*p* = 0.002, *t* = 3.70, *df* = 9). For three-way diagnosis, the best cluster-based Diagnostic Pipeline achieved 66.0 ± 5.1% accuracy. Comparison of this accuracy with the baseline chance accuracy of 64.2 ± 3.8% approached significance (*p* = 0.07, *t* = 1.59, *df* = 9). This Diagnostic Pipeline used SWA with reduction factor *r* = 8, scan-based percent signal change intensity normalization (PSCS-s), PCA_1−5,Cluster_ feature extraction based on comparison of ADHD patients vs. controls as well as ADHD-C vs. ADHD-I patients, and the cubic SVM classifier. Compared to this cluster-based approach, the best personal characteristic-based Diagnostic Pipeline (section 3.1) achieved an accuracy of 69.0 ± 8.3%, and the comparison of these accuracies approached significance (*p* = 0.08, *t* = 1.55, *df* = 9).

**Figure 4:**
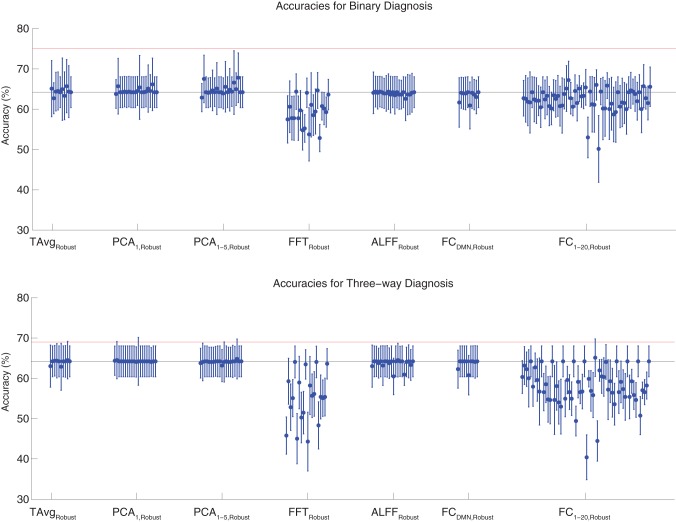
**Top**: Accuracies on the binary diagnosis task (controls vs. ADHD) using features derived from the robust cluster identification procedure (section 2.6) with five different classifiers (section 2.2). Each blue dot with error bars shows the mean accuracy and standard deviation for one Diagnostic Pipeline (feature data and classifier combination) on 10-fold cross validation with the 668 participant Training Dataset. The black horizontal line at 64.2% accuracy indicates chance baseline (guessing healthy control for all participants). The red horizontal line at 75.0% accuracy indicates the best mean accuracy achieved with binary diagnosis on the Training Dataset using personal characteristic input data (section 3.1). **Bottom**: Accuracies on the three-way diagnosis task (controls vs. ADHD-C vs. ADHD-I) using the same Diagnostic Pipelines as in **top panel**. The red horizontal line at 69.0% accuracy indicates the best mean accuracy achieved with three-way diagnosis on the Training Dataset using personal characteristic input data (section 3.1). Other conventions as in **top panel**.

Binary and three-way diagnosis were also tested with feature extraction methods utilizing the robust cluster extraction procedure. These feature extraction methods included the T Avg_Robust_, PCA_1,Robust_, PCA_1−5,Robust_, FFT_Robust_, ALFF_Robust_, FC_DMN,Robust_, and FC_1−20,Robust_ procedures (see section 2.6). See Figure 4 for results. None of these tests performed better than the best Diagnostic Pipelines using personal characteristic data (section 3.1).

For binary diagnosis, the best robust cluster extraction Diagnostic Pipeline achieved 67.8 ± 6.2% accuracy, which was significantly better than the baseline chance accuracy of 64.2 ± 3.8% (*p* = 0.03, *t* = 2.23, *df* = 9). This Diagnostic Pipeline used SWA with reduction factor *r* = 8, scan-based percent signal change intensity normalization (PSCS-s), PCA_1−5,Robust_ feature extraction based on comparison of ADHD patients vs. controls as well as ADHD-C vs. ADHD-I patients, and the cubic SVM classifier. In comparison to this robust cluster-based method, the best personal characteristic-based Diagnostic Pipeline (section 3.1) achieved a significantly better accuracy of 75.0 ± 4.5% (*p* = 0.006, *t* = 3.18, *df* = 9).

For three-way diagnosis, the best robust cluster extraction Diagnostic Pipeline achieved 65.1 ± 4.7% accuracy. This was not significantly better than the baseline chance accuracy of 64.2 ± 3.8% though it approached significance (*p* = 0.07, *t* = 1.62, *df* = 9). This Diagnostic Pipeline used FC_1−20,Robust_ feature extraction based on comparison of ADHD patients vs. controls and the RBF SVM classifier. In comparison to this robust cluster-based approach, the best personal characteristic-based Diagnostic Pipeline (section 3.1) achieved a significantly better accuracy of 69.0 ± 8.3% (*p* = 0.03, *t* = 2.09, *df* = 9).

### 3.4. Control experiment: training and testing on the same data

As a control experiment, Diagnostic Pipelines were also trained and then tested on the same training data. Many of the Diagnostic Pipelines whose test accuracies are shown in Figures 2–4 obtained perfect accuracies (100 ± 0%) or very good accuracies (above 90%) on the training data. For example, the FC_1−20,Cluster_ feature set (section 2.6) yielded 100 ± 0.0% accuracy on the training set for three-way diagnosis with the logistic, quadratic SVM, and cubic SVM classifiers. This feature set included 200 features per participant (i.e., feature vector length was 200), which was well below the number of participants (668) used for 10-fold cross validation. All Diagnostic Pipelines using FC_1−20,Cluster_ features performed at or below chance when tested on the test data. We can conclude that there is diagnostically-useful information in the fMRI data and that some Diagnostic Pipelines were able to utilize this information at least for the data on which they were trained. These Diagnostic Pipelines were not able to generalize well to new test data, resulting in performance at or below baseline chance accuracy in most cases and only 6.5% better than chance in the best case.

### 3.5. fMRI functional connectivity group analyses

**Figure 5:**
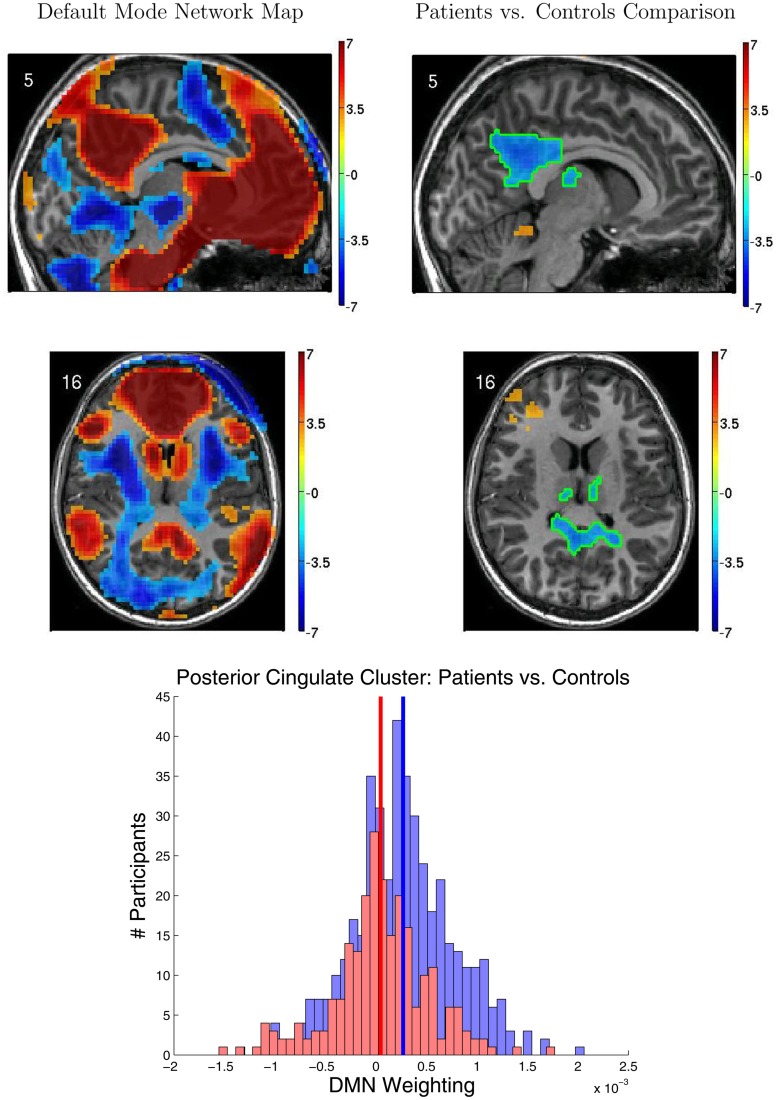
**Results from functional connectivity group analysis. Top-left**: A midline sagittal slice from a statistical map of voxels with significant weighting for the default mode network (DMN) in 668 participants. Red regions were positively weighted on the DMN. Blue regions were negatively weighted. Front of brain is on the right side of the image. Colored bar indicates *t*-value scaling. The slice's coordinate in mm in MNI space is shown in the upper-left corner. All results *p* < 0.05 corrected for multiple comparisons. **Middle-left**: Axial slice of same DMN weighting map as shown in upper-left panel. Left side of brain is on left side of image. Other conventions as in top-left panel. **Top-right**: Sagittal slice from a contrast map comparing DMN weighting for 239 ADHD patients vs. 429 healthy controls. Red regions showed greater DMN weighting in patients, whereas blue regions showed greater weighting in controls. One cluster of significant difference, including parts of posterior cingulate cortex and thalamus, is outlined in green. Other conventions as in top-left panel. **Middle-right**: Axial slice from same contrast map as shown in upper-right panel. Conventions as in middle-left panel. **Bottom**: Histograms of patients' (red bars) and controls' (blue bars) average DMN weighting values across the voxels in the posterior cingulate/thalamus cluster which is outlined in green on the contrast maps. Group means are shown as thick vertical bars (red: patients, blue: controls). Though mean DMN weighting was significantly larger for controls than for patients in this region, there was substantial overlap among the weighting values for the participants' in the two groups.

Figure 5 shows results from the FC group analyses, including the DMN localizer map and the statistical contrast map comparing DMN weighting for ADHD patients vs. healthy controls. The DMN included medial prefrontal cortex, posterior cingulate cortex and precuneus, and the temporal-parietal junction. We found significantly greater DMN weighting for controls vs. ADHD patients (*p* < 0.05 corrected) in posterior cingulate cortex, bilateral anterior superior temporal sulcus, and bilateral thalamus. ADHD patients exhibited greater DMN weighting than controls in left anterior middle frontal gyrus, right temporal pole, and superior cerebellum. Though these regions exhibited group differences between controls and patients, there was substantial overlap among participants from the two groups (Figure 5 bottom).

**Figure 6:**
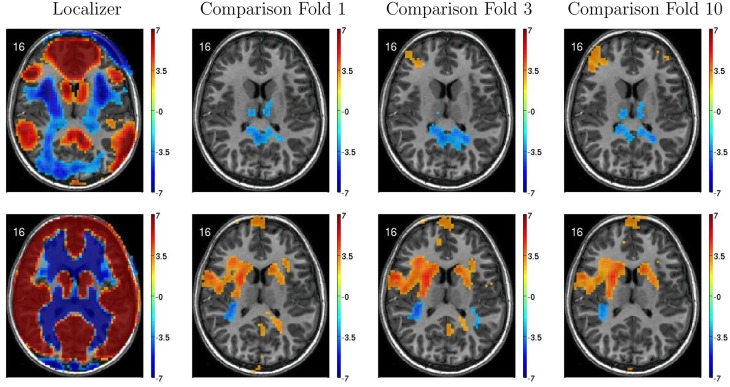
**Top-row**: Axial slices of default mode network (DMN) localizer map and three patients vs. controls statistical comparison maps computed on different folds of 10-fold cross validation. Each fold omitted from the comparison a different subset of 66 or 67 participants out of the 668 participant Training Dataset. For the localizer map, red/blue regions exhibited weighting significantly above/below zero. For contrast maps, red and blue regions exhibited greater weighting for patients and for controls, respectively. Left side of axial slice represents left side of brain. Numbers in the upper-left corners of the images indicate slice coordinates in MNI space. Color bars show *t*-value scaling. **Bottom-row**: Equivalent to **top-row** expect that localizer and contrast maps were taken from a different functional connectivity (FC) weighting map. This map appears to reflect gray matter and white matter structure. It is noteworthy that most but not all clusters of significance were present in all three-folds depicted. In addition, cluster sizes and shapes differed to some extent on different folds.

Figure 6 illustrates variability in results of group comparisons between ADHD patients and controls depending on which subsets of participants were used in the comparisons. For two different FC weighting maps (one of which was the DMN map), Figure 6 shows contrast maps computed on three different folds of 10-fold cross validation. That is, each contrast map was computed omitting a different subset (containing 66 or 67 participants) of the 668 participant Training Dataset. Though the maps generated on different folds were for the most part qualitatively similar, with clusters of significant difference in equivalent locations, the precise sizes and shapes of the clusters differed. In addition, some significant clusters were not present in all maps. These differences may have important implications for generalizing group differences to between-individuals analyses such as diagnostic testing.

## 4. Discussion

Twenty-one teams took part in the ADHD-200 Global Competition. Our personal characteristic-based diagnostic approach outperformed all imaging-based approaches from the other 20 competing teams (see ADHD-200-Results-Webpage, 2011) in addition to our own imaging-based tests. The best-performing imaging-based method was submitted by the Johns Hopkins University team, whose accuracy on three-way diagnosis was 60.51%, in comparison to our personal characteristic-based diagnostic accuracy of 62.52% on the same data. Our results highlight the importance of accounting for personal characteristics like age, gender, IQ, and the site of data collection in studies of fMRI-based diagnosis. In particular, it is important to include control tests of diagnosis using only personal characteristic input.

The relative success of our diagnostic method in the absence of fMRI input generated some discussion online (SMART, 2011; Yarkoni, 2011a,b). Poldrack pointed out that the utility of personal characteristic variables for diagnosis suggests that “any successful imaging-based decoding could have been relying upon correlates of those variables rather than truly decoding a correlate of the disease” (see Yarkoni, 2011b). The SMART working group (Point 4 of SMART, 2011) has pointed out that high diagnostic utility is a demanding criterion for any input feature to achieve and that lack thereof does not imply an absence of biological significance for the feature in question. We agree with this point. For the current discussion, we will focus on the application of machine learning using personal characteristic and fMRI inputs, working toward delivering clinical diagnostic tools.

### 4.1. Challenges of fMRI-based diagnosis in large datasets

The best accuracies achieved by the ADHD-200 competition teams were 62.52% using personal characteristic data and 60.51% using fMRI data. For comparison, one would obtain a chance accuracy of 55.0% by guessing “healthy control” for all participants in the ADHD-200 Holdout Dataset (Original Holdout Dataset). Previous studies have reported successes with fMRI-based diagnosis of ADHD (Zhu et al., 2005, 2008) and other psychiatric illnesses (Shinkareva et al., 2006; Calhoun et al., 2008; Fu et al., 2008; Marquand et al., 2008; Cecchi et al., 2009; Arribas et al., 2010; Nouretdinov et al., 2011; Shen et al., 2010; Costafreda et al., 2011; Fan et al., 2011). These twelve studies reported diagnostic accuracies ranging from 68% to 89% with a median of 86%. Superficially, these observations may suggest that it is fairly challenging to diagnose ADHD with high accuracy in the ADHD-200 sample. The ADHD-200 dataset presented unique and novel challenges for fMRI-based diagnosis. As we discuss below, this dataset is probably much closer to the data that would be generated in real-world clinical settings, in comparison to the more constrained datasets used in previous studies. We argue that achieving three-way diagnostic accuracies better than chance with this dataset is an encouraging early success in this context.

Firstly, the ADHD-200 competition requirement for three-way diagnosis is intrinsically more challenging than the binary diagnosis considered in most previous studies. The standard accuracy score is computed as the number of participants correctly classified/total number of participants. This score favors binary diagnosis, which differentiates patients vs. controls, over diagnosis with three or more diagnostic classes. In three-way diagnosis, participants correctly classified as patients but with the wrong diagnosis count as complete misses when computing accuracy scores. For example, this happens when an ADHD patient is classified as having ADHD but with the wrong ADHD subtype. Accordingly, our best binary diagnostic accuracy of 75.0% on the Training Dataset (see Table 1) was substantially better than our best three-way diagnostic accuracy of 69.0% on the same data. It would be informative in future fMRI diagnosis competitions to have a binary diagnosis sub-competition in which competitors attempt to differentiate simply between patients and healthy controls.

Secondly, the ADHD-200 competition forced competitors to use a Holdout Dataset that could not be used for training classifiers nor for selecting a classifier algorithm, feature extraction procedure, or preprocessing procedure. This is in contrast to some previous studies which tested multiple classifiers without correcting for multiple comparisons (over-fitting). For example, suppose one were to test a dozen different classifiers using *n*-fold cross validation to protect against over-fitting within each test. By emphasizing the results from the best-performing algorithm or reporting only those results from the best algorithm, one still exposes oneself to the multiple comparison problem by virtue of testing multiple classification algorithms. The best performing algorithm may be best for the particular dataset being used for testing, simply by chance. That algorithm may not generalize to new data drawn from the same distribution. The ADHD-200 competition results were based on a holdout dataset. Using a holdout set avoided the multiple comparison problem and predisposed the ADHD-200 results to compare unfavorably to studies that did not correct for multiple comparisons. We see this effect in our tests of personal characteristics-based diagnosis. The Diagnostic Pipelines that performed best on our Training Dataset achieved lesser accuracy when tested on our Holdout Dataset (see section 3.1 and Tables 3 and 4).

Thirdly, the ADHD-200 dataset presented new challenges in terms of participant heterogeneity with which previous studies did not have to contend. The ADHD-200 sample contains 973 participants. This is many more participants than were included in the previous studies cited above. Those twelve studies included from 14 to 104 participants with a median of 39 participants. In a study with 20 participants, it is possible to select them to be fairly homogeneous in terms of age, gender, socio-economic background, medication history, and clinical sequelae. In contrast, the ADHD-200 sample pooled hundreds of participants from eight different sites in various countries. We expect that the ADHD-200 data were more heterogeneous compared to datasets used in previous fMRI diagnosis studies. Combining data from multiple sites introduced additional potential heterogeneity in terms of participant nationality, genetic background, and cultural background, as well as potential differences in how clinical groups in different institutions applied ADHD diagnostic criteria. MRI scanner hardware and settings certainly differed across the eight sites contributing to the ADHD-200 dataset, in contrast to previous studies that collected all data on a single scanner. Previous studies of fMRI-based diagnosis did not have to contend with heterogeneity on this scale. The ADHD-200 sample is the first fMRI dataset to combine such large numbers of psychiatric patients and control participants. That the ADHD-200 Global Competition teams did not achieve high accuracy diagnosis with this data may simply reflect the need for new methodological approaches to address this heterogeneity. More research into new fMRI-based diagnostic methods that are robust against participant heterogeneity is clearly warranted and may enable high accuracy diagnosis with the ADHD-200 dataset in the future. Because data from clinical settings also vary across sites, scanners, patients populations, and so on, robustness against these sources of data heterogeneity will be important as we work toward deploying fMRI-based diagnosis in clinical settings.

To reduce inter-site variability, we tested diagnosis using resting state fMRI data from ADHD-200 participants all taken from a specific site. Within-site diagnosis was not more successful on average than using the entire Training Dataset (results not shown). Our robust cluster-based feature extraction methods were an attempt to improve diagnostic performance generalization to novel test data by reducing feature heterogeneity, but these attempts were unsuccessful. Most of the individual ADHD-200 sites included relatively large numbers of participants, for example 216 from NYU. The data from a single site likely still exhibited substantial heterogeneity among participants, and the diagnostic algorithms we tested may not have been robust against this heterogeneity.

### 4.2. Challenges for fMRI-based diagnosis of ADHD

The ADHD-200 Global Competition teams did not achieve high accuracy diagnosis using the resting state fMRI scans in the ADHD-200 sample. It is possible that resting state fMRI may not be an ideal imaging protocol for diagnosing ADHD specifically. Before adopting this strong conclusion, one would obviously need to exclude the possibility that the ADHD-200 competition teams may have used non-optimal diagnostic approaches, as discussed above. One other group has reported 85% accuracy on binary diagnosis of ADHD patients vs. controls using resting state fMRI data (Zhu et al., 2005, 2008). These studies both utilized the same group of 9 ADHD patients and 11 controls. It is likely that these small participant groups were more homogeneous than the ADHD-200 participant groups. It is unknown whether Zhu et al. (2005, 2008)'s diagnostic methods will scale to larger, more heterogenous participant groups. To explore this question, we are currently in the process of implementing Zhu et al. (2005, 2008)'s diagnostic methods to test them on the ADHD-200 sample. It is possible that fMRI data with a different psychological task, such as an attention task or response inhibition task, may be more effective for fMRI-based diagnosis of ADHD. To our knowledge, no one has attempted diagnosis of ADHD with these fMRI protocols.

We must also consider that ADHD may be generally more difficult to diagnose with fMRI than other psychiatric illnesses. There is ongoing controversy over whether ADHD is over-diagnosed (for example, see Bruchmueller et al., 2012). The presence of individuals incorrectly diagnosed as having ADHD in the patient group would partially invalidate the “ground truth” diagnostic labels that a machine learning classifier attempts to learn and reproduce. Such incorrect ground truth labels could cause a machine learning algorithm to miss diagnostically-useful patterns in the data, thereby making it harder to learn an effective diagnostic classifier.

### 4.3. Group vs. individual differences

We found significant differences in FC patterns between patients and controls. Specifically, involvement in the DMN (Raichle et al., 2001; Fox et al., 2005) differed between patients and controls in several regions including posterior cingulate cortex. We know of one study (Qiu et al., 2011) that compared ADHD patients with controls using resting state fMRI and an ICA-based analysis similar to ours. Other studies (Cao et al., 2006, 2009; Tian et al., 2006, 2008; Zang et al., 2007; Castellanos et al., 2008; Uddin et al., 2008; Wang et al., 2009; Fair et al., 2010; Liston et al., 2011; Sun et al., 2012) used very different FC analyses, which means we cannot directly compare their results with ours. Qiu et al. (2011) found lesser DMN involvement for ADHD patients compared to controls in posterior cingulate, as well as differences in several regions where we did not find differences.

**Figure 7:**
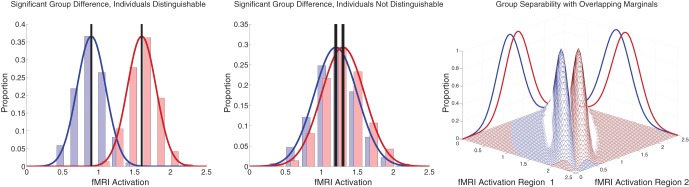
**Illustration of group differences vs. individual differences. Left** and **middle** panels show simulated fMRI activation levels from an arbitrary brain region for two groups (blue and red) of 1000 participants each. Frequency histograms for participants' activation levels appear as pale blue and pale red bars. The actual Gaussian distributions from which participants were drawn are shown as dark blue and dark red curves. Black vertical rectangles (which may appear as thick black lines) show 95% confidence intervals of average activation across each group. Group comparisons between blue and red average activation levels are significant in both panels (**left**: *p* « machine precision, *t* = 78.7, *df* = 1998; **middle**: *p* = 2 × 10^−12^, *t* = 7.1, *df* = 1998). The high statistical significance in both cases derives from the large number of participants, which allows us to estimate the means with high precision (Central Limit Theorem). There is little overlap between the blue and red groups in the **left panel**, and activation levels can predict individuals' groups with accuracy of 93%. In the **middle panel**, there is substantial overlap between the groups, and inferring participants' groups from their activation levels yields poor accuracy of 56% (compared to baseline chance accuracy of 50%). This illustrates that group differences with high statistical significance can, but do not necessarily, translate into good diagnostic criteria for distinguishing individuals from different groups. **Right panel** shows that combining activation levels from two regions, neither of which can separate the groups on its own because the marginal distributions overlap substantially, can allow good separation of participant groups if the joint distributions do not overlap much.

Between-group differences identified in group analyses can potentially, but do not necessarily, distinguish the individual participants that comprise different groups. Even when two groups differ significantly in the mean, there can be substantial overlap among the individuals in the two groups, making classification difficult (illustrated in Figure 7 left and middle panels). Also see Friston (2012). This was the case with our FC group analysis. There was substantial overlap among ADHD patients' and controls' DMN weighting values in regions that exhibited highly significant differences between the groups (Figure 5 bottom). Combining features that are individually poor for classification into higher dimensional feature vectors can potentially allow one to separate individuals by group. Figure 7 right panel shows a very simple version of this idea. Many of the Diagnostic Pipelines incorporating FC input features were able to detect diagnostically-meaningful patterns in the fMRI data, which allowed them to achieve very high or even perfect accuracies when they were trained and then tested on the same participants' data. (This was the case even with feature vector lengths less than the number of participants.) These Diagnostic Pipelines still did not generalize well to new data—they performed poorly on novel test participants. We propose that this poor generalization performance was due to variability in the diagnostic patterns extracted from the fMRI data during classifier training with different subsets of participants. This proposition is supported by the variability we observed in the FC analysis group comparisons when we varied which subsets of the participant population were used for the comparisons (Figure 6). Though these comparisons yielded qualitatively similar results, there was variability in precisely which voxels exhibited significant differences and, thus, variability in the precise locations, shapes, and sizes of clusters of significant difference. These observations motivate future work on improved methods for extracting more reliable differences in fMRI data with greater out-of-sample validity. One potential avenue of investigation might focus less on traditional group comparison analyses and more on individual differences, for example by adapting various machine learning algorithms to fMRI applications.

## 5. Conclusions

The ADHD-200 Global Competition presented the first opportunity to test fMRI-based diagnosis of a psychiatric illness with a large dataset comprised of hundreds of participants from multiple data collection sites. Encouragingly, the ADHD-200 competition teams achieved three-way diagnostic accuracies above chance on this challenging dataset. The relative success of using personal characteristics such as age, gender, IQ, and site of data collection to perform diagnosis indicates that such personal characteristic data should be considered carefully in fMRI-based diagnostic work as well as in studies employing group comparisons. It is important to include control tests of diagnosis based only on personal characteristic data. Combining personal characteristic data and fMRI data has the potential to improve diagnostic accuracy (see Sidhu et al., 2012, under review). Between-groups statistical comparisons did not produce features (biomarkers) capable of diagnosing individual participants with high accuracy. Because the ADHD-200 dataset combined large numbers of participants from different data collection sites, it imposed novel challenges in terms of inter-participant heterogeneity. Future studies of fMRI-based diagnosis must develop methods that are robust against such heterogeneity. One potential approach may be to adapt existing machine learning methods, which were developed in other contexts, to the identification of individual differences in fMRI datasets. Such individual differences may have better diagnostic utility than differences derived from group comparisons. Novel methods that are robust against heterogeneity will be particularly important as we work toward delivering new fMRI-based clinical diagnostic tools that could be deployed in diverse, real-world health care settings.

### Conflict of interest statement

The authors declare that the research was conducted in the absence of any commercial or financial relationships that could be construed as a potential conflict of interest.

## Figures and Tables

**Figure A1:**
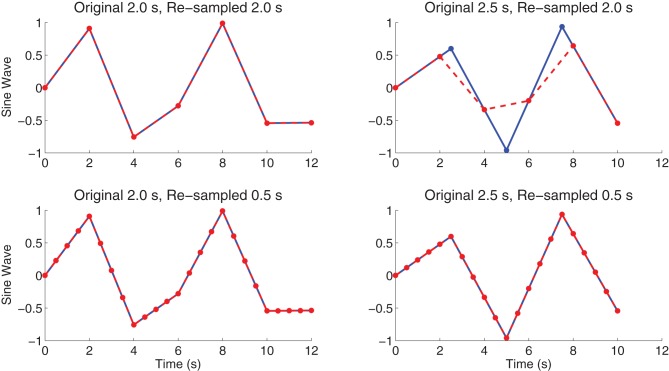
**Figure shows sine waves (blue) with original sampling periods of 2.0 or 2.5 s as well as re-sampled curves (red) with re-sampling periods of 2.0 or 0.5 s.** Red curves were linearly interpolated from blue curves. Re-sampling from 2.0 to 2.0 s (**upper-left panel**) leaves the curve unchanged. Re-sampling from 2.5 to 2.0 s (**upper-right panel**) causes re-sampling errors—the minimum and maximum values for the red curve are closer to zero than for the blue curve. Linear re-sampling from 2.0 or 2.5 s into 0.5 s (**lower-left** and **lower-right** panels) does not introduce such re-sampling errors, though of course it cannot recover sine wave values for time points falling in-between the originally-sampled time points.

**Figure A2:**
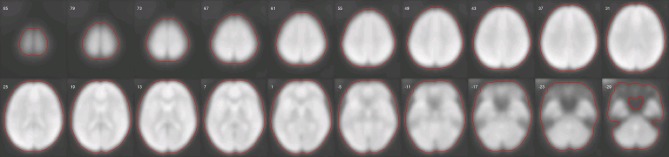
**Figure shows slices from the between-subject mean-normalized fMRI volume (see text).** Red line indicates the manually-determined edge of the binary mask volume.

## Appendix

### A. Array notation

For the discussion that follows, we define some notation for 3D arrays. Let **V** be a 3D array. Typically, **V**'s first, second, and third axes will correspond, respectively, to the left-right, posterior-anterior, and inferior-superior axes of the brain (as in the NIFTI data format standard). **V**_(*i*,*j*,*k*)_ is the intensity value of the voxel at index position (*i*, *j*, *k*) counting from zero. For example, **V**_(0,0,0)_ is the intensity of the voxel in the left-most, bottom-most, back-most corner of the scan volume. **V**_(9,19,29)_ is the intensity of the voxel which is 10th from the left side of the scan, 20th from the back, and 30th from the bottom. **V**_(*a* → *b*, *c* → *d*, *e* → *f*)_ is the 3D sub-array of **V** going from index *a* to *b* (inclusive) on the first axis, from *c* to *d* on the second axis, and from *e* to *f* on the third axis. Size(**V**) is a vector of length equal to the number of axes of **V** containing integer values denoting the number of elements in **V** along each axis. If **V** is a 57 × 67 × 50 array, Size(**V**) = [57,67,50]^*T*^. Size(**V**)_*i*_ is the number of elements in **V**'s axis *i*, counting from 0. We can then write Size(**V**) = [Size(**V**)_0_, Size(**V**)_1_, Size(**V**)_2_]^*T*^. The modulus Mod(*x*, *y*) where *x* and *y* are integers is the integer remainder after dividing *x* by *y* [e.g., Mod(7,4) = 3, Mod(11,3) = 2]. Fix(*x*,*y*) where *x* and *y* are integers is *x* divided by *y* and then rounded toward zero [e.g., Fix(7,4) = 1, Fix(11,3) = 3]. **V_FLAT_** is the column vector produced by flattening **V**. **V_FLAT_**_*i*_ is the *i*'th element of **V_FLAT_** counting from zero. Letting *s*_*i*_ = Size(**V**)_*i*_,

(1) $$\begin{array}{c} V_{FLAT}{}_{i}=V_{(\text{Mod}(i,s_{0}),}\\ {}_{\text{Fix}(\text{Mod}(i,s_{0}\times s_{1}),s_{0}),}\\ {}_{\text{Fix}(i,s_{0}\times s_{1}))} \end{array}$$

(This is equivalent to **V**(:) if **V** were a 3D array in MATLAB.)

### B. Temporal resampling

In the ADHD-200 dataset, the resting state fMRI scans collected from the various sites had different volumes times (sampling periods) including 1.5 s (88 participants), 1.96 s (48 participants), 2.0 s (410 participants), 2.5 s (221 participants), and 3.0 s (1 participant). fMRI data preprocessing step 6 (see section 2.4) included up-sampling each scan into a 0.5 s volume time (2 Hz sampling rate) using linear interpolation. We chose to up-sample, rather than re-sampling into an intermediate sampling period such as 2.0 s, to minimize temporal re-sampling errors. If we had re-sampled all scans into a 2.0 s volume time, those scans originally sampled at 1.5, 2.5, and 3.0 s would have been affected by interpolation errors (see Figure A1). Up-sampling into a 0.5 s volume time avoided this problem because the original volume times are integer multiples of 0.5 s. (1.96 s is not an exact integer multiple of 0.5 s, but it is close. It would not have been practical to use a re-sampled volume time of 0.02 s, which is the largest integral divisor of all the original volume times.)

### C. Intensity normalization

Given a voxel's unnormalized (raw) timecourse vector **x_raw_**_*i*_, the percent signal change scaled (PSCS-tc) version of that timecourse is defined as:

(2) $$x_{i}=100\times (x_{raw}{}_{i}-\text{Mean}(x_{raw}{}_{i}))/\text{Mean}(x_{raw}{}_{i}),$$

where Mean(**x_raw_**_*i*_) is the mean over the timecourse **x_raw_**_*i*_.

Given an unnormalized (raw) 4D scan **X_raw_** and unnormalized voxel timecourse vector **x_raw_**_*i*_, percent signal chance scaling based on the scan mean (PSCS-s) was defined as:

(3) $$x_{i}=100\times (x_{raw}{}_{i}-\text{Mean}(X_{raw}))/\text{Mean}(X_{raw}),$$

where Mean(**X_raw_**) is the mean over the entire 4D scan **X_raw_**.

### D. Spatial window averaging

SWA reduced the spatial dimensionality in a fashion somewhat similar to down-sampling. Let reduction factor *r* be an integer with *r* ≥ 1. Consider a 3D volumetric array **V**. We imposed a 3D grid over the volume with grid spacing = *r*. We computed the average intensity over the *r*^3^ voxels within each grid box (window). These values constituted a new 3D array **V_reduced_** with volume 1/*r*^3^ the original. Partial grid windows could occur at the edges of the volume if *r* was not an integer factor of one or more of the original **V**'s axis lengths Size(**V**). In these cases, we truncated the original **V** evenly on all sides so as to fit the grid without partial boxes on the edges.

(4) $$\begin{array}{c} V_{reduced}{}_{\left(i,j,k\right)}=\text{Mean}(V_{((ir+{\text{offset}}_{0})\to (ir+(r-1)+{\text{offset}}_{0}),}\\ {}_{(jr+{\text{offset}}_{1})\to (jr+(r-1)+{\text{offset}}_{1}),}\\ {}_{\left(kr+{\text{offset}}_{2})\to (kr+(r-1)+{\text{offset}}_{2})\right)}) \end{array}$$

where offset_*i*_ = Fix(Mod(Size(**V**)_*i*_, *r*), 2). By applying SWA to every volume (time point) of a participant's 4D fMRI data array, we spatially reduced the entire 4D array, leaving the temporal dimension unchanged. We used either *r* = 8 yielding a reduced fMRI data spatial size of 8 × 7 × 6 voxels, *r* = 3 yielding a reduced fMRI data spatial size of 19 × 22 × 16 voxels, or *r* = 1 resulting in no dimensionality reduction. We implemented SWA in custom MATLAB code.

### E. Masking

We used a binary mask to remove voxels outside the brain (Figure A2). A binary mask volume **M** was built as follows. For each of the 668 Training Dataset participants, the first volume (first time point) of the participant's resting state fMRI scan was mean-normalized—every voxel's intensity was divided by the mean intensity across the whole volume. We then took the mean volume across the 668 mean-normalized volumes and thresholded it using a manually-determined threshold of 0.5 intensity to create a binary mask volume with values of 1 for voxels inside the brain in most participants and values of 0 for voxels outside the brain in most participants. The mask had the same spatial dimensions as participants' fMRI data (57 × 67 × 50). The mask marked 97,216 voxels as being inside the brain out of a total of 190,950 voxels.

The masking procedure mapped a 3D volume **V** of size 57 × 67 × 50, representing one fMRI time point, to a column vector **V_masked_** of size 97,216 × 1. Recall from Appendix A that **V_FLAT_** is the column vector produced by flattening **V** and **V_FLAT_**_*i*_ is the i'th element of **V_FLAT_** counting from zero. **V_masked_** was derived by concatenating the values **V_FLAT_**_*i*_ for all *i* where **M_FLAT_**_*i*_ = 1.

The masking procedure for an individual volume was extended to 4D fMRI scan data as follows. Let **A** be a 4D array of size 57 × 67 × 50 × 370 representing an fMRI scan composed of 370 volumes (times points) **V**_*t*_. The masked version of **A** was a matrix **A_masked_** produced by applying the masking procedure to each **V**_*t*_ and then concatenating the resulting **V_masked_**^*T*^_*t*_ row vectors. We will call such a matrix representing a masked fMRI scan **X**.

(5) $$X=A_{masked}=\left[\begin{array}{c} V_{masked}{}_{0}^{T}\\ V_{masked}{}_{1}^{T}\\ \ldots \\ V_{masked}{}_{369}^{T} \end{array}\right]$$

To combine SWA with masking with a given reduction factor *r* (see above), we applied SWA to the full size binary mask volume to create a reduced size mask volume. SWA on a binary volume creates voxels with scalar values between 0 and 1. Such values were thresholded, replacing intensities greater than or equal to 0.5 with 1 and values less than 0.5 with 0. We then proceeded with masking on the SWA-reduced fMRI data set using the reduced mask.

### F. Principal components analysis

Let **X**_*S*__*i*_ be an *n*_timepoints_ × *n*_voxels_ matrix representing the fMRI scan from participant *i* after SWA with reduction factor *r* = 8 and masking (see Appendix E.) Let **D** be the *n*_timepoints_ × (*n*_voxels_ × *n*_participants_) matrix produced by horizontally concatenating all **X**_*S*__*i*_:

(6) $$D=\left[X_{1}X_{2}\ldots X_{n_{\text{participants}}}\right].$$

A centered version of the data matrix **D_c_** was produced by subtracting the row mean from each row of **D**. For our PCA dimensionality reduction, we computed the eigenvectors and eigenvalues of the covariance matrix **D_c_D**^*T*^_**c**_/(*n*_voxels_ × *n*_participants_ − 1). We ranked the eigenvectors in descending size of their corresponding eigenvalues and retained either the first eigenvector (PCA_1_) or the first five eigenvectors (PCA_1−5_). For each participant *i*, the participant's data **X**_*s*__*i*_ were projected onto the one or five eigenvectors resulting in an *n*_eigenvectors_ × *n*_voxels_ matrix **P**_*S*__i_. The flattened version of this matrix **P_FLAT_**_*S*__*i*_ was the feature vector for participant *i*.

### G. Feature normalization

Features were normalized using Weka's min./max. standardization. (Note: Weka uses “normalization” to mean Z-scaling and “standardization” to mean the min./max. normalization described here.) Let the classifier input data be represented by an *n*_participants_ × *n*_features_ matrix **F** with each row consisting of one participant's feature vector. Each column *c*_*i*_ of **F** contained feature element *i* for all participants. Each such column was normalized thus:

(7) $$c_{i,\text{normalized}}=(c_{i}-\text{MIN}(c_{i}))/(\text{MAX}(c_{i})-\text{MIN}(c_{i}))$$

Also see Witten et al. (2011).

### H. Cluster extraction

Let **T** be a statistical parametric T map—that is, a 3D array representing the *T*-values resulting from performing a *t*-test at each voxel location in an fMRI data set. **T** is assumed to have been locally thresholded such that |**T**| > θ_*t*_ for all voxels, where θ_*t*_ might be 2.0 for example. The *d*_min_ parameter is the minimum interpeak distance in mm. The *v*_min_ parameter is the desired minimum cluster size in mm^3^. The *n*_clusters_ parameter is the maximum number of clusters to return. The cluster extraction algorithm performed two passes, one for positive parts of the map and one for the negative parts. On a given pass, the algorithm located the statistical peak locations (local extrema) and ranked them. If *d*_min_ > 0, the algorithm iterated through the peaks in descending order, discarding those peaks less than *d*_min_ mm away from the nearest peak with a larger |**T**| value. Each surviving peak served as the seed for a cluster. The clusters were grown in parallel by iteratively adding to a given cluster any unassigned, non-zero-valued voxels that were the immediate neighbor of a voxel already in the cluster. In the case of a conflict where a voxel neighbored onto two growing clusters, the cluster with the higher peak (seed) value won. After completion of the growth process, any cluster with volume less than *v*_min_ was absorbed into the neighboring (abutting) cluster with the highest peak (seed) value if possible. If no such neighboring cluster was available, a small cluster was left as it was. In addition, the algorithm checked for “orphaned” voxels that no growing cluster could reach because the requisite seed voxel was removed in the minimum inter-peak assertion process at the start. In such cases, the required peak voxel was re-instated and a new cluster grown around it to absorb unassigned voxels. This algorithm split large contiguous regions of statistical significance that contained several local statistical peaks into multiple clusters. Regions of statistical significance with only one peak tended to be smaller regions, and these regions were assigned to one cluster. Finally, all regions were ordered by absolute statistical mass, that is, the sum of the |**T**| values for all voxels in a cluster. The *n*_clusters_ clusters with the largest absolute statistical mass were returned. Setting *d*_min_ = 0 mm, *v*_min_ = 0 mm^3^, and *n*_clusters_ = ∞ caused this algorithm to return the same output as MATLAB's bwlabel function.

### I. Cluster size thresholding

Let **T** be a statistical parametric T map that was locally thresholded such that |**T**| > θ_*t*_ for some threshold parameter θ_*t*_. A set of clusters *c* was extracted from **T** using automated cluster extraction (see Appendix H) with *d*_min_ = 0, *v*_min_ = 0, and *n*_clusters_ = ∞. For every cluster *c*_*i*_ in *c*, if *c*_*i*_ had fewer voxels than the minimum cluster size θ_cs_, all voxels in **T** contained in cluster *c*_*i*_ had their values set to zero.
